# Naringin Reduces Hyperglycemia-Induced Cardiac Fibrosis by Relieving Oxidative Stress

**DOI:** 10.1371/journal.pone.0149890

**Published:** 2016-03-11

**Authors:** Olubunmi A. Adebiyi, Oluwafeyisetan O. Adebiyi, Peter M. O. Owira

**Affiliations:** Molecular and Clinical Pharmacology Research Laboratory, Department of Pharmacology, Discipline of Pharmaceutical Sciences, School of Health Sciences, University of KwaZulu-Natal, P.O. Box X5401, Durban, South Africa; University of Buenos Aires, Faculty of Medicine. Cardiovascular Pathophysiology Institute., ARGENTINA

## Abstract

**Introduction:**

Hyperglycemia promotes myocardial fibrotic lesions through upregulation of PKC and p38 in response to redox changes. The effects of naringin on hyperglycemia-induced myocardial fibrotic changes and its putative effects on PKC-β and p38 protein expression in type 1 rat model of diabetes are hereby investigated.

**Methods:**

Male Sprague-Dawley rats were divided into six groups I-VI. Groups I and II, were orally treated with distilled water {3.0 ml/kg body weight (BW)} and naringin (50 mg/kg BW), respectively. Groups III, IV, V and VI were rendered diabetic by a single intraperitoneal injection of streptozotocin (60 mg/kg, BW) and were similarly treated with subcutaneous insulin (8.0 I.U/kg BW, twice daily), naringin (50 mg/kg BW), distilled water (3.0 ml/Kg BW) and ramipril (3.0 mg/kg/BW), respectively. The animals were sacrificed after 56 days by halothane overdose; blood and heart samples removed for further analysis.

**Results:**

The untreated diabetic rats exhibited significantly increased oxidative stress, NADPH oxidase activity, increased cardiac fibrosis, PKC-β and p38 mitogen activated protein kinase expression compared to controls. Naringin treatment significantly ameliorated these changes in diabetic rats compared to the untreated diabetic controls.

**Conclusions:**

Naringin’s amelioration of myocardial fibrosis by modulating p38 and PKC-β protein expression possibly through its known antioxidant actions and may therefore be useful in retarding the progression of fibrosis in a diabetic heart.

## Introduction

Myocardial fibrosis (MF) is a distinct structural feature of cardiac remodelling and a common finding in the histopathological examinations of the myocardium and a predisposing risk factor for the development of heart failure in patients in type 1 diabetes mellitus (T1DM) [1–3]. MF is a consequence of hyperglycemia-induced exacerbation of extracellular matrix (ECM) remodelling characterised by increased deposition of ECM components, impairment of collagen lysis, cardiac fibroblast proliferation and the accompanying myocardial stiffness which interfere with normal cardiac functioning [1, 4–6].

Persistent hyperglycemia also promotes MF through direct activation of cardiac fibroblast proliferation, indirect activation of NADPH oxidase activity secondary to increased angiotensin II production and increased oxidative stress [2, 3, 7, 8]. Angiotensin II exerts its profibrotic effects by activating cardiac fibroblasts to produce ET-1 (Endothelin 1); a vasoactive peptide (whose activity and expression of its receptors are elevated in diabetic hearts) and through increased activation of cardiomyocyte membrane bound NADPH oxidase activity which is associated with increased generation of reactive oxygen species [3]. Furthermore, increased activity of NADPH oxidase in diabetic hearts has been reported and the reactive oxygen species (ROS) consequently produced have been strongly linked to the development of myocardial fibrosis through the activation of matrix metalloproteinases and increased fibroblast proliferation [9–11]. It is evident from the recent experimental data that oxidative stress plays a pivotal role in ECM remodelling in the diabetic heart and most of the ROS responsible for this enhanced matrix remodelling is suggested to be sourced from NADPH oxidase [12–14]. Therefore, the restoration of redox balance appears to be an important consideration in the prevention or treatment of myocardial fibrosis in diabetes mellitus. Hyperglycemia pro-fibrotic effects are also associated with the upregulation and activation of signaling molecules including protein kinase C (PKC) and mitogen activated kinases such as c-Jun Nuclear Kinase (JNK), p38 and extracellular kinase related kinase 1 (ERK1/2) [3]. PKC and p38 activation have been shown to contribute significantly to the development of myocardial fibrosis and adverse remodeling of the diabetic heart [3]. Furthermore, there is evidence supporting the contribution of increased ROS generation from NADPH oxidase in the activation or up regulation of PKC and p38α in the myocardium [3].

Currently, treatment options for myocardial fibrosis are limited and efforts in the management of cardiac fibrosis are focused on the reversal of fibrosis in a bid to improve overall cardiac function and survival [2]. It has been proposed that strategies that either reduce ROS or augment myocardial antioxidant defence mechanism are likely to be efficacious in improving myocardial function in diabetes due to the central role that oxidative stress plays in the pathogenesis of MF [3, 15]. Therefore, antioxidants could be potential therapeutic agents in the prevention of the development and progression of ROS-induced adverse myocardial changes associated with diabetes mellitus [3]. Naringin (4,5,7-trihydroxyflavanone-7-rhamnoglucoside) is a bio-flavonoid that is derived from grapefruit and related citrus species and has been reported to possess potent antioxidant/free radical scavenging properties [16, 17], anti-apoptotic activity [18, 19] hypoglycaemic effects in type 2 DM [20, 21, 22, 23] and anti-inflammatory effects [24]. Potential cardioprotective properties of naringin have previously been suggested [16, 25] but its efficacy in oxidative-stress induced myocardial fibrosis in the context of type 1 diabetes mellitus is unknown. We therefore postulated that naringin through its known antioxidant effects could reverse or ameliorate hyperglycaemia-induced oxidative stress and possibly limit or reverse myocardial fibrosis in a diabetic heart.

## Materials and Methods

### Chemical Reagents

All chemicals used in these experiments unless otherwise stated were purchased from Sigma-Aldrich^®^ Chemicals (St. Louis, Montana, USA)

### Experimental Animals

Male Sprague-Dawley rats (235± 15 g) supplied by the Biomedical Resource Unit (B.R.U) of the University of KwaZulu-Natal, Durban, South Africa were housed in well ventilated transparent standard plastic rat cages at the animal holding unit of the B.R.U. One week was allowed for acclimatization of the animals to their new environment before the study was commenced. The animals were maintained at temperature of 23–25°C, humidity 55–60% and 12 hour daylight/dark cycle throughout the study period and were fed standard commercial rat chow with free access to drinking water ad libitum. The study had institutional approval from the Biomedical Research Ethics Committee (BREC) of the University of KwaZulu-Natal (008/BREC/14). Strict adherence to institutional protocols and guidelines of the BREC on the care and handling of animals were ensured.

### Induction of Type 1 Diabetes Mellitus

Diabetes was induced in the rats by a single intraperitoneal injection of 60 mg/kg BW streptozotocin (STZ) dissolved in 0.2 mL of 0.1 M freshly prepared citrate buffer (pH 4.5) after an overnight fast. The non-diabetic controls were similarly treated with vehicle. Development of diabetes was confirmed 3 days later by measuring Fasting Blood Glucose (FBG) via tail prick sampling using a standard glucometer (one Touch select, Milpitas, CA, USA). Rats with post-STZ FBG levels greater than 8.0 mmol /L were considered diabetic and included in the study.

### Experimental Design and Procedure

Age and weight matched rats were randomly divided into six groups (n = 7). Groups I and II were orally treated daily with 3.0 ml/kg body weight (BW) and 50 mg/kg of distilled water and naringin, respectively while groups III-VI were diabetic. Groups III, IV and VI were treated with Actraphane insulin (4 I.U/kg BW twice daily, s.c) (Norvodisk^®^, Norway), oral naringin (50 mg/kg BW/day) and oral ramipril (3.0 mg/kg BW/day) (Novartis^®^, South Africa), respectively. Group V was left untreated and served as the diabetic control group. At the start of experiments baseline FBG measurements were determined in all rats. The live body weights and water intake of the rats were measured daily and recorded throughout the duration of the study. On day 54 of the study, the rats were placed in metabolic cages and 24-hour urine was collected and recorded. The Glucose Tolerance Tests (GTT) were carried out on day 56 after the rats were fasted overnight and baseline FBG levels determined prior to the intraperitoneal administration of 3.0 g/kg BW of glucose-D in normal saline. Blood glucose concentrations were measured at times 0, 15, 30, 60, 120 minutes in all treatment groups. Areas Under the Curve (AUC) were calculated from blood-glucose time curves (mmol/L x time in mins) and presented as AUC units [26]. On day 57, the rats were euthanized by halothane overdose. Blood samples were collected by cardiac puncture and the separated plasma was stored at -70°C for further biochemical analysis. The hearts were quickly excised, washed in ice-cold Phosphate Buffered Saline (PBS), blotted dry and weighed. The left ventricles were quickly dissected free and weighed. The tissues were subsequently rapidly snap frozen in liquid nitrogen and stored at -70°C for further analysis.

### NADPH Oxidase Activity Assay

Left ventricular cardiac tissue NADPH Oxidase-dependent superoxide production was measured by Cytochrome C Reduction assay as previously described [27]. Fifty milligram of fresh left ventricle tissue was homogenized in 450 μL of ice-cold-buffer containing 50 mM KPHO_4_ pH 6.7 and 1.0 mM EDTA. Protein content of the homogenate was measured by the Bradford method [28]. Two hundred microliters of tissue homogenate (final concentration 1.0 mg/mL) diluted in Dulbecco’s modified Eagle’s medium (Lonza^®^, Germany) without phenol red was aliquoted into each well of a 96-well microplate to a final concentration of 1.0 mg/ml. Into each well was added 25 μL of 500 μM Cytochrome C and 25 μL of 100 μM NADPH in the presence or absence of 10 μL superoxide dismutase (200 U/mL) and the plate was incubated at room temperature for 30 minutes with intermittent agitation. Cytochrome C reduction was measured by reading absorbance at 550 nm in nanostar^®^ Microplate reader (California, USA). Superoxide production (in nmol/mg protein) was calculated from the difference between absorbance with and without superoxide dismutase and extinction coefficient for change of ferricytochrome c to ferrocytochrome c, i.e., 21.0 mmol L^−1^ cm^−1^ [29]. Calculated NADPH oxidase activity was then expressed as nmol/mg of protein.

### Advanced Oxidation Protein Products (AOPP) Assay

Plasma and tissue AOPP content were determined as per the modified method of Witko-Servasat et al, [30]. Briefly, 50 mg of ventricular tissue was homogenized in 450 μL of ice cold buffer containing 50 mM KPHO_4_ pH 6.7 and 1.0 mM EDTA and the homogenate was centrifuged at 10,000 g at 4°C for 10 minutes. Into the test wells of the 96-well microplate, 200 μL of plasma or supernatant diluted 1/5 in PBS were added followed by 10 μL of 1.16 M potassium iodide and 20 μL of acetic acid. Into standard wells, 200 μL of chloramine-T solution (0–100 μM), 10 μL of 1.16 M potassium iodide followed by 20 μL of acetic acid were added. AOPP was measured by spectrophotometry on a Microplate reader (Nanostar^®^, California, USA) at 340 nm against a blank containing 200 μL of PBS, 10 μL of potassium iodide, and 20 μL of acetic acid. The concentrations of AOPP in samples were expressed as micromoles per liter of chloramine-T equivalents.

### Cytosolic Superoxide Dismutase (SOD) Activity Assay

The CuZnSOD activities were assayed using commercially available ELISA kit (Cayman chemicals, USA) according to the manufacturer’s instructions. The cytosolic SOD activities were determined by measuring absorbances of test samples at 450 nm in a Micro-plate reader (Spectrostar, California USA). The CuZnSOD activities were calculated as the amount of SOD that produced a 50% dismutation of superoxide anions and were expressed in U/ml.

### Masson Trichrome Stain

Left ventricular sections fixed in 10% neutral buffered formalin were embedded in paraffin. They were deparaffinised and rehydrated through graded alcohol series followed by a wash in distilled water. The sections were stained successively in Weigert’s iron haematoxylin solution, Bierbrich scarlet-acid fuchsin, phosphomolybdic-phosphotungstic acid solution and aniline blue solution. Thereafter, the sections were rapidly dehydrated through 95% ethyl alcohol/ absolute ethyl alcohol and were cleared in xylene. The sections were mounted with resinous mounting medium. The sections were scanned on the Olympus compound microscope X451 (Hitachi, Japan) and analysed using NIS-300 software (Version 3). Histological analysis of area of fibrosis were done at x400 magnification using 10 fields per slide (n = 4). Results were expressed as a percentage of the total field area. The fibrotic areas were identified by color contrast and fibrotic areas within each field were measured, computed and divided by the total field area and expressed as a fraction of 100%.

### Protein Kinase C-β and p38 MAPK Expression

The Western immunoblot assay was used in detecting the expressions of PKC-β1 and p38α protein in cardiac tissue. Left ventricular tissue (100 mg) were homogenised and centrifuged at 12,000 x g at 4°C for 20 minutes and the protein content were determined by Bradford method [28]. Twenty micrograms of samples were resolved on a 10% SDS page gel for 90 minutes at 150 V and transferred to nitrocellulose membranes in a wet transfer system at 100 V for 1 hour. The membranes were blocked by incubating in Tris-Buffered saline containing 1% Tween 20 (TBS-T) containing 3% Bovine serum Albumin (BSA) for 1 hour at room temperature. The membranes were subsequently incubated with rabbit polyclonal PKC-β1 (1:200) and p38 antibodies (1:200) (Santa Cruz biotechnology, Santa Cruz, USA), respectively overnight at 4°C. The membranes were then washed (X3) with TBS-T before incubation with Horse-Radish peroxidase (HRP) conjugated goat anti-rabbit secondary antibody (1:5,000 dilution), (Santa Cruz biotechnology, Santa Cruz, USA) for 1.0 hour. After three further washes with TBS-T solution, the membranes were developed with Lumiglo^®^ chemilumiscent reagent (Cell signalling Inc) and visualized on the Chemi-Doc^®^ (Biorad, Hercules, USA). The relative densities of the bands were analysed with in-built image lab software.

### Statistics

Data were presented as mean ± SEM. Statistical analyses were determined using either unpaired student t-test or One Way Analysis of Variance (ANOVA) followed by multiple comparison Student Newman Kuels test using a Graphpad^®^ statistical software (version 5). A p-value of less than 5% (p<0.05) was considered statistically significant.

## Results

### Confirmation of Type 1 Diabetes

**Fig 1:**
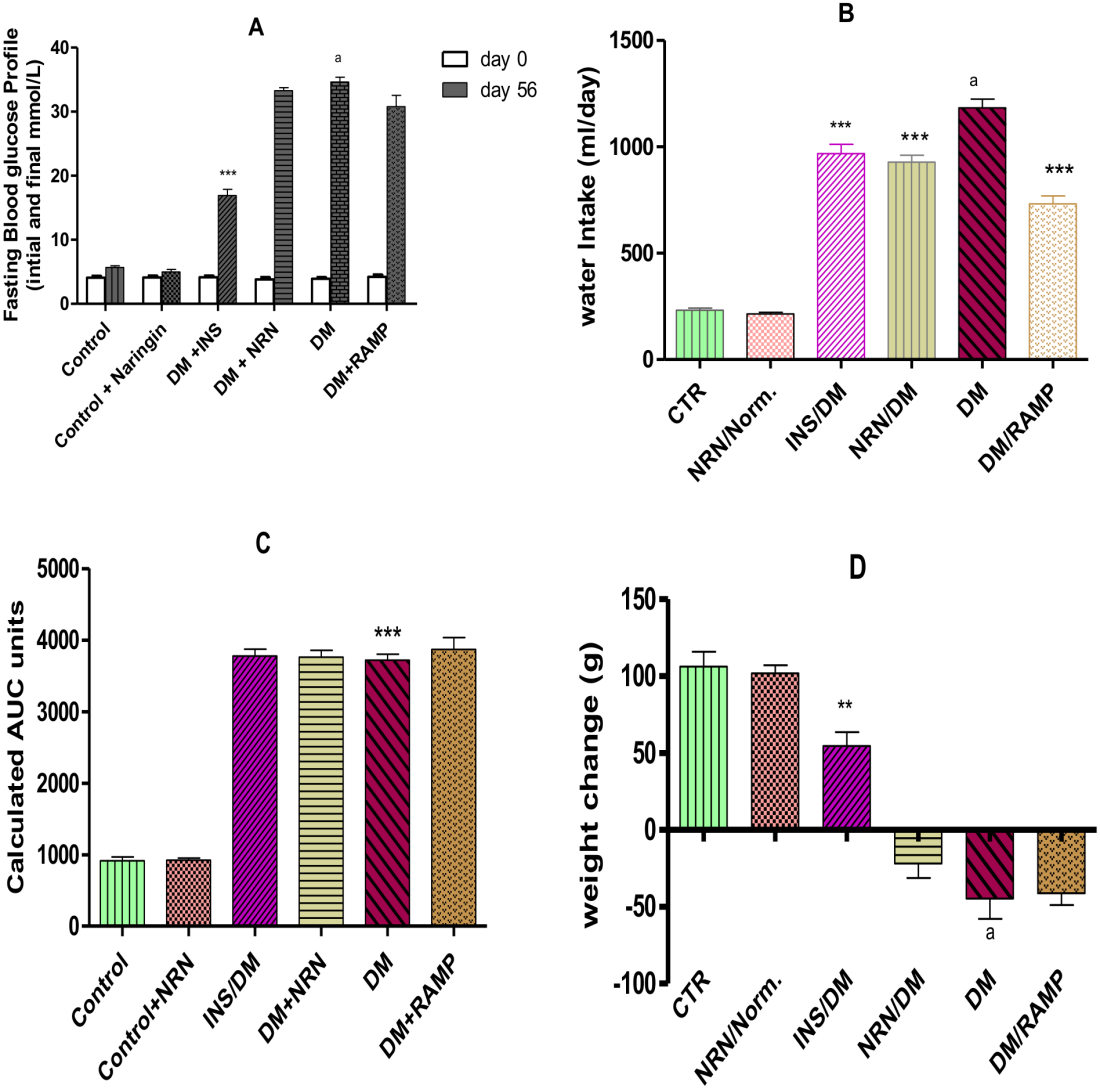
Confirmation of diabetes mellitus **A**: FBG before induction of diabetes (day 0) and at the end of the study (8 weeks post-diabetes induction). [***p<0.0001(compared to DM day 56) and ^a^p<0.0001 (compared to control day 56)]. **B**: Water consumption (Volume in mls obtained by deducting final volume from initial volume of water) in 56 days. [***p<0.001 (compared to DM) and ^a^p<0.0001 (compared to control)] **C**: AUC calculated from GTT-time curves. [***p<0.001 (compared to controls]. **D**: Differences in live weight between initial weight (day 0) and final weight (day 56). [**p<0.001 (compared to DM) and ^a^p<0.0001 (compared to control)].CTR (control), NRN (Naringin), NRN (Naringin), INS (Insulin), DM (Diabetes Mellitus) RMP (Ramipril).

Diabetic rats exhibited significantly (p<0.05) higher FBG levels compared to the vehicle treated controls (Fig 1A). Insulin treated group showed significantly (p<0.05) lower FBG compared to the untreated diabetic rats. However, naringin and ramipril treated groups did not show significant changes in their FBG levels relative to the untreated diabetic rats. Furthermore, the untreated diabetic rats showed significantly (p<0.05) increased polydipsia compared to the non-diabetic controls (Fig 1B). However, naringin, ramipril and insulin treatment of the diabetic rats significantly (p<0.05) reduced polydipsia compared to the untreated diabetic rats. Also, untreated diabetic rats showed markedly significant (p<0.01) glucose intolerance compared to the vehicle-treated controls but neither naringin nor ramipril treatment significantly alleviated glucose intolerance in diabetic rats compared to diabetic untreated rats (Fig 1C). Furthermore, the untreated diabetic rats had significant (p<0.05) weight losses compared to the vehicle treated controls (Fig 1D). However, insulin treatment of diabetic rats significantly (p<0.05) prevented weight loss compared to the untreated diabetic rats. Naringin treated diabetic rats on the other hand did not exhibit significant reversal of weight loss compared to the untreated diabetic rats.

### NADPH Oxidase Activity

**Fig 2:**
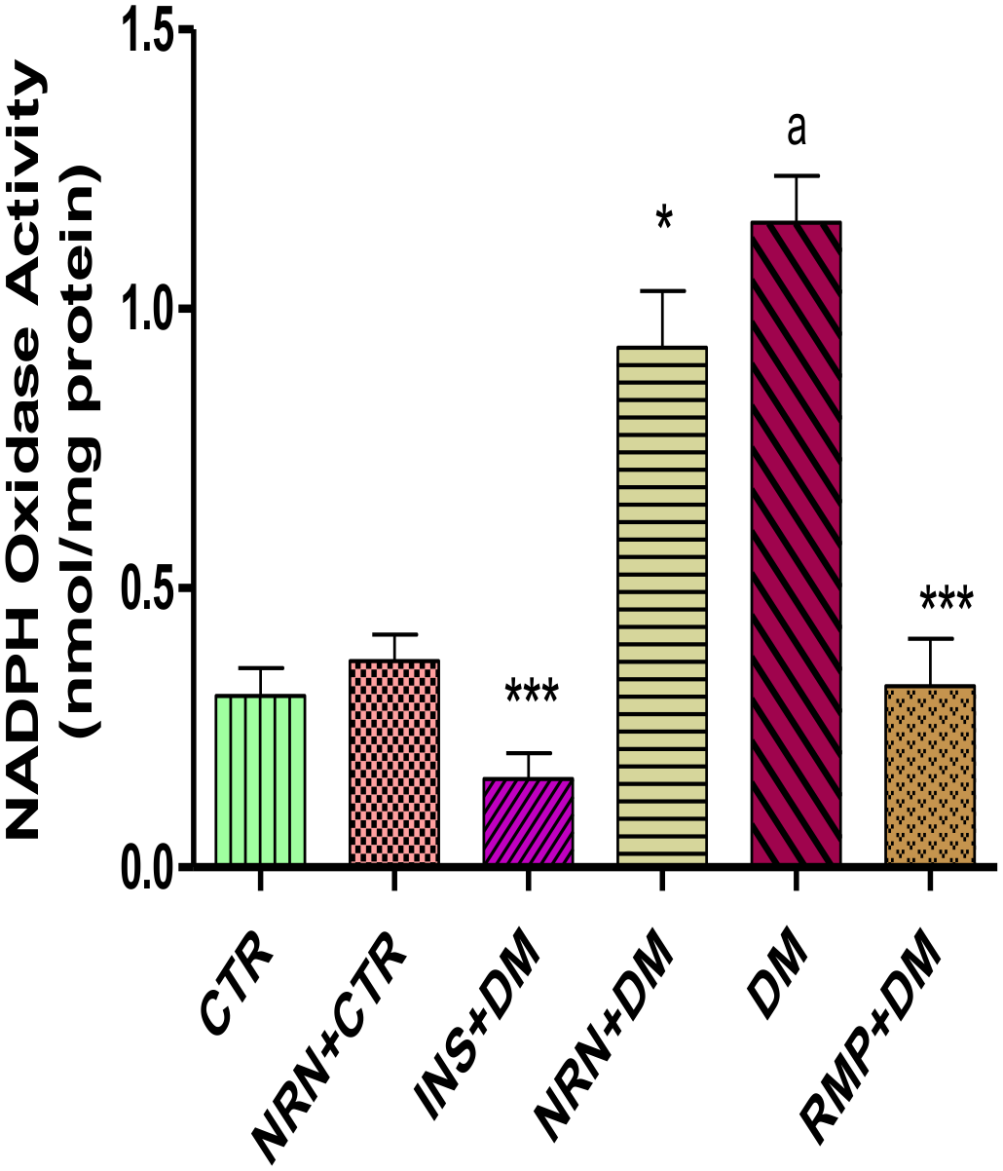
Cardiac tissue NADPH Oxidase activity. [***p<0.001, *p<0.05 (compared to DM) and ^a^p<0.0001 (compared to CTR)]. CTR (control), NRN (Naringin), NRN (Naringin), INS (Insulin), DM (Diabetes Mellitus) RMP (Ramipril).

The NADPH oxidase activities in untreated diabetic rats were significantly (p<0.0001) increased compared to the vehicle treated controls (Fig 2). However, treatment of the diabetic rats with naringin, insulin and ramipril significantly (p<0.05) reduced the activity of NADPH oxidase relative to the untreated diabetic rats.

### Cytosolic Superoxide Dismutase (CuZnSOD) Activity

**Fig 3:**
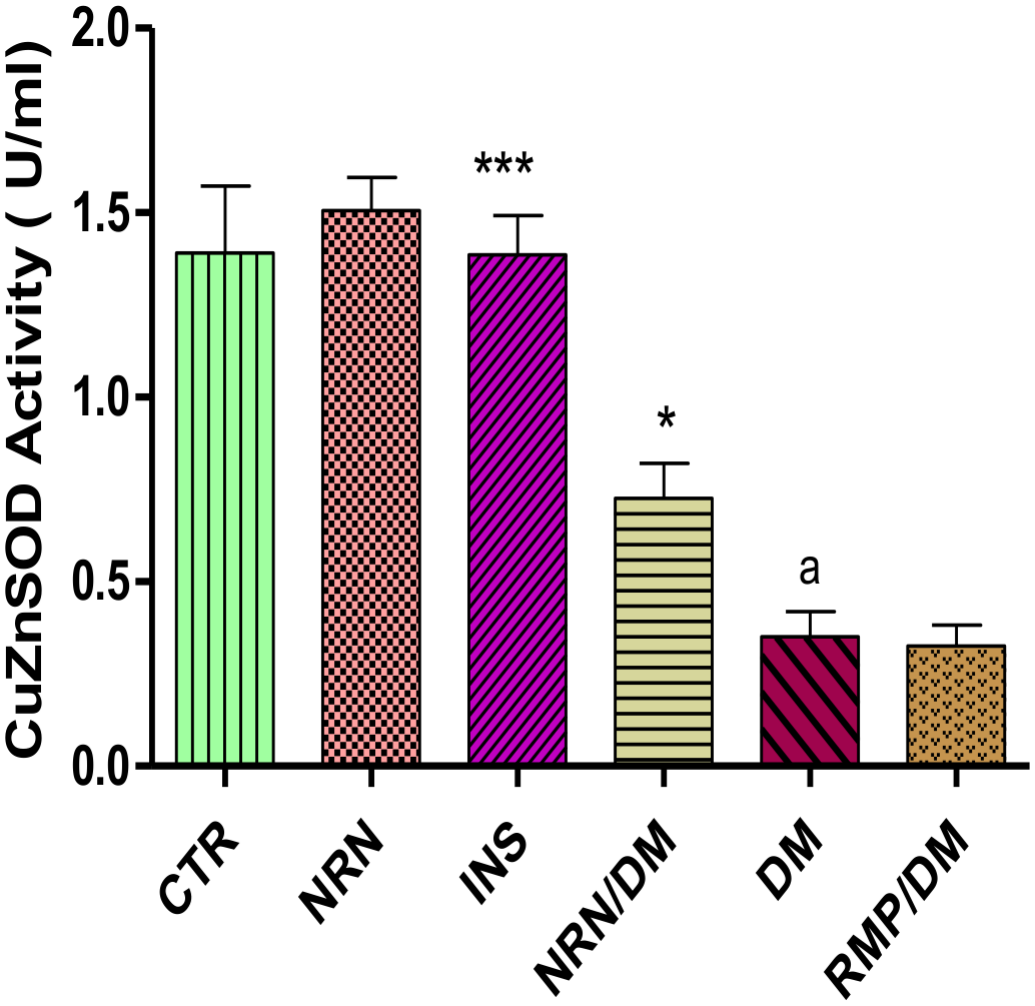
Cytosolic myocardial SOD (CuZnSOD) activity. [***p<0.001, *p<0.05 (compared to DM) and ^a^p<0.0001 (compared to CTR)].CTR (control), NRN (Naringin), NRN (Naringin), INS (Insulin), DM (Diabetes Mellitus) RMP (Ramipril).

The activities of the cytosolic SOD (CuZnSOD) in the myocardium of the untreated diabetic rats were significantly (p<0.05) reduced by 67% in comparison to the non-diabetic controls (Fig 3). Naringin and insulin but not ramipril treatments of diabetic rats significantly (p<0.05) increased CuZnSOD activity by 65% and 200%, respectively compared to the untreated diabetic controls.

### Plasma and Cardiac Advanced Oxidation Protein Products (AOPP)

**Fig 4:**
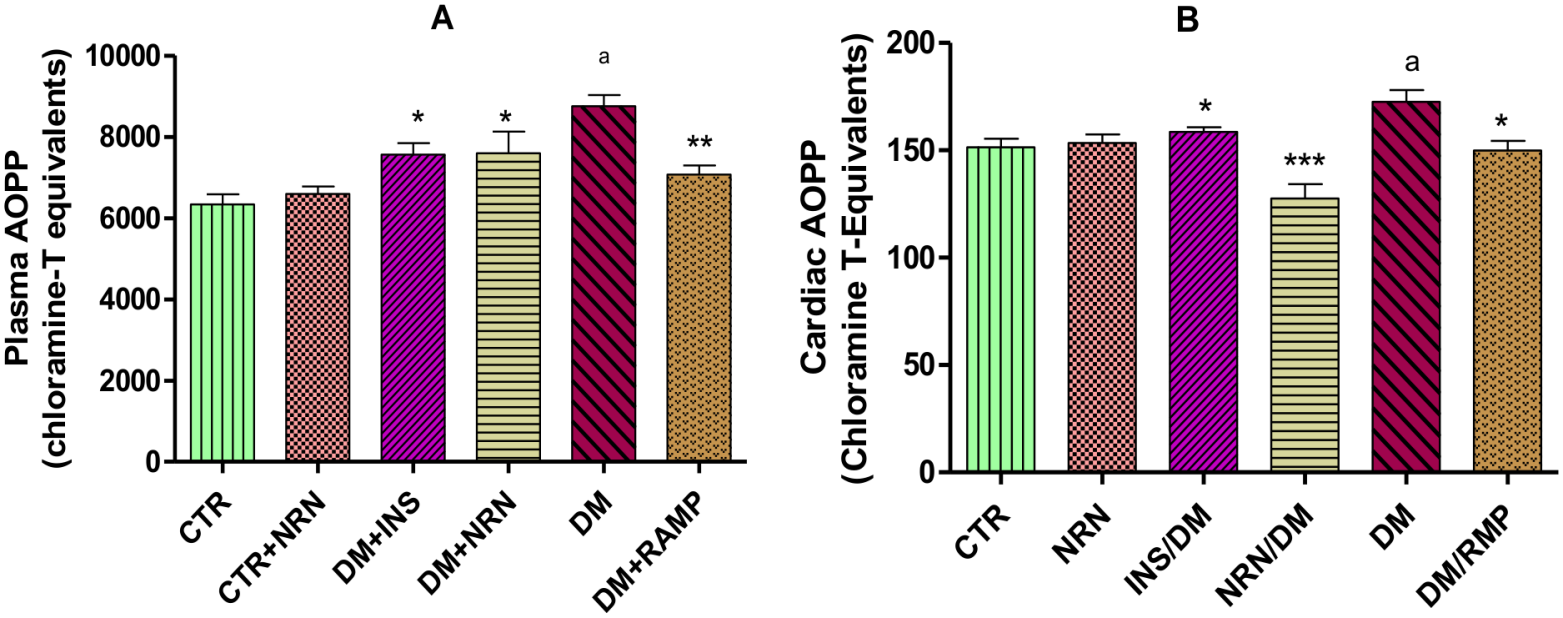
A: Plasma and B: cardiac Advanced Oxidation Protein Products (AOPP). Control (CTR), naringin (NRN), diabetes mellitus (DM), ramipril (RAMP), insulin (INS) [*p<0.05, **p<0.01 and ***p<0.0001 (compared to DM), ^a^p<0.05 (compared to CTR)].

Plasma and cardiac AOPP in the untreated diabetic rats were significantly (p<0.05) elevated compared to the non-diabetic controls (Fig 4A and 4B). However, the naringin-, insulin- and ramipril-treated diabetic rats exhibited significant (p<0.05) reduction in both the plasma and cardiac AOPP relative to the untreated diabetic controls, respectively.

### Myocardial Fibrosis

**Fig 5:**
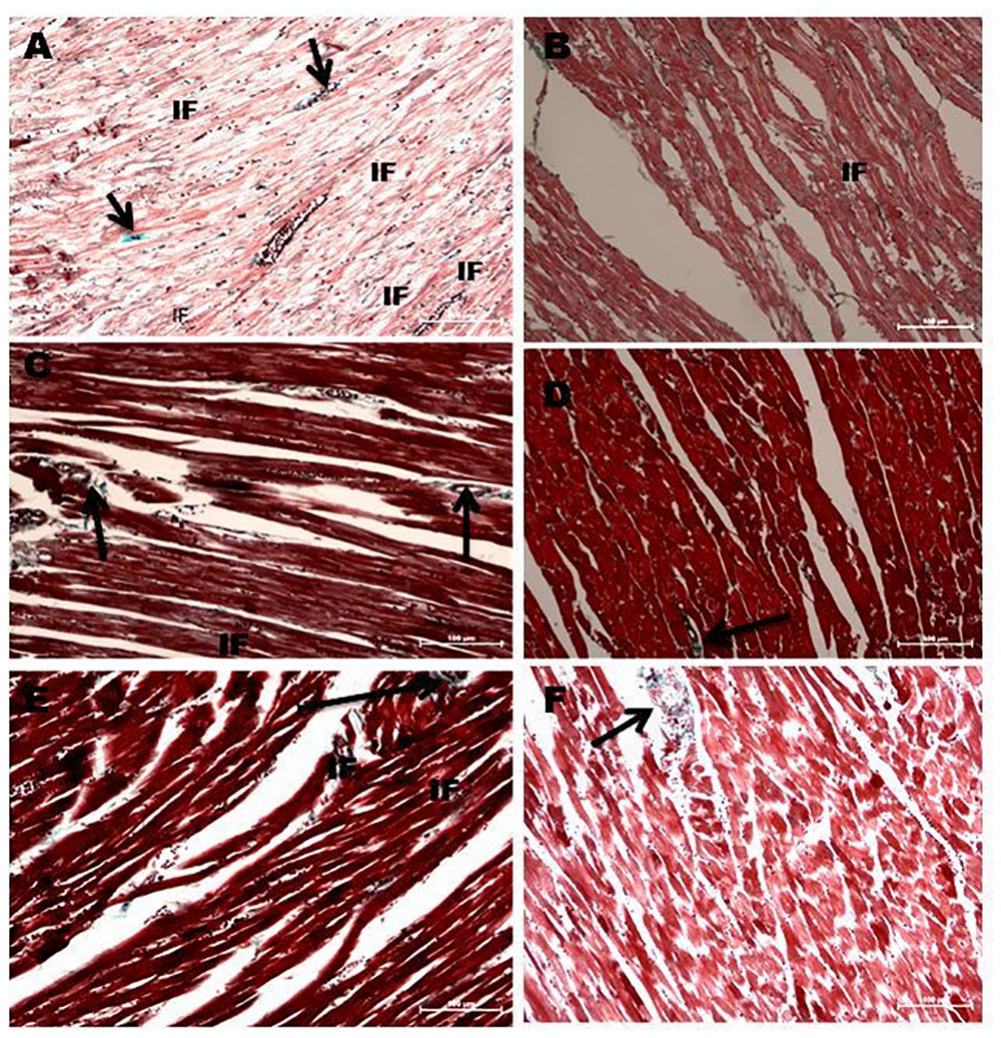
**A**: Light Microscopy studies using Masson Trichrome stain showing longitudinal section of left Ventricular Myocardium of Diabetic rats (X200). Black arrows indicate perivascular concentrations of fibrotic tissue while the “**IF**” arrows show areas of interstitial fibrosis. **A**: Diabetic untreated rats **B**: Naringin- treated diabetic rats. **C**: Ramipril-treated diabetic rats. **D**: Naringin-treated normal rats. **E**: Insulin-treated diabetic rats **F**. Control rats.

**Fig 6:**
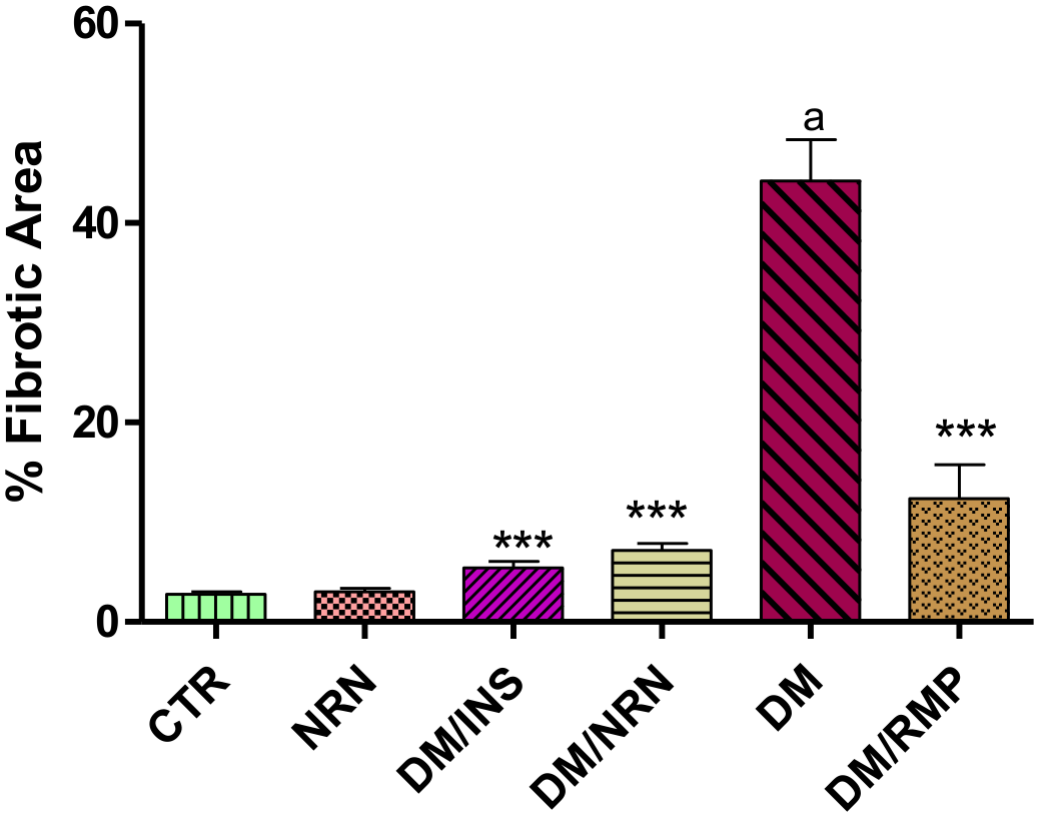
Calculated percentage fibrotic area. The fibrotic areas were identified by color contrast in the NIS-300 software and calculated as a percentage of the total field area. Ten fields per slide were analysed (n = 4). [***p<0.0001 (compared to DM), ap<0.05 (compared to CTR)]. Control (CTR), naringin (NRN), diabetes mellitus (DM), insulin (INS), ramipril (RMP).

The untreated diabetic rats showed increased interstitial and perivascular location of fibrotic scars compared to the vehicle-treated controls (Fig 5A and 5F). However, naringin, ramipril and insulin treatment of diabetic rats reduced the interstitial fibrosis and the perivascular fibrotic changes compared to the untreated diabetic rats (Fig 5B, 5C and 5E). The untreated diabetic rats showed significantly (p<0.001) increased percentage area of fibrosis compared to vehicle-treated non-diabetic controls (Fig 6). However, treatment of diabetic rats with naringin profoundly and significantly (p<0.001) reversed the increase in the percentage fibrotic areas in diabetic rats compared to the untreated diabetic rats. Ramipril and insulin produced comparable and significant reduction in the percentage fibrotic areas compared to the untreated diabetic controls.

### Protein Kinase C and p38 MAPK Expressions

**Fig 7:**
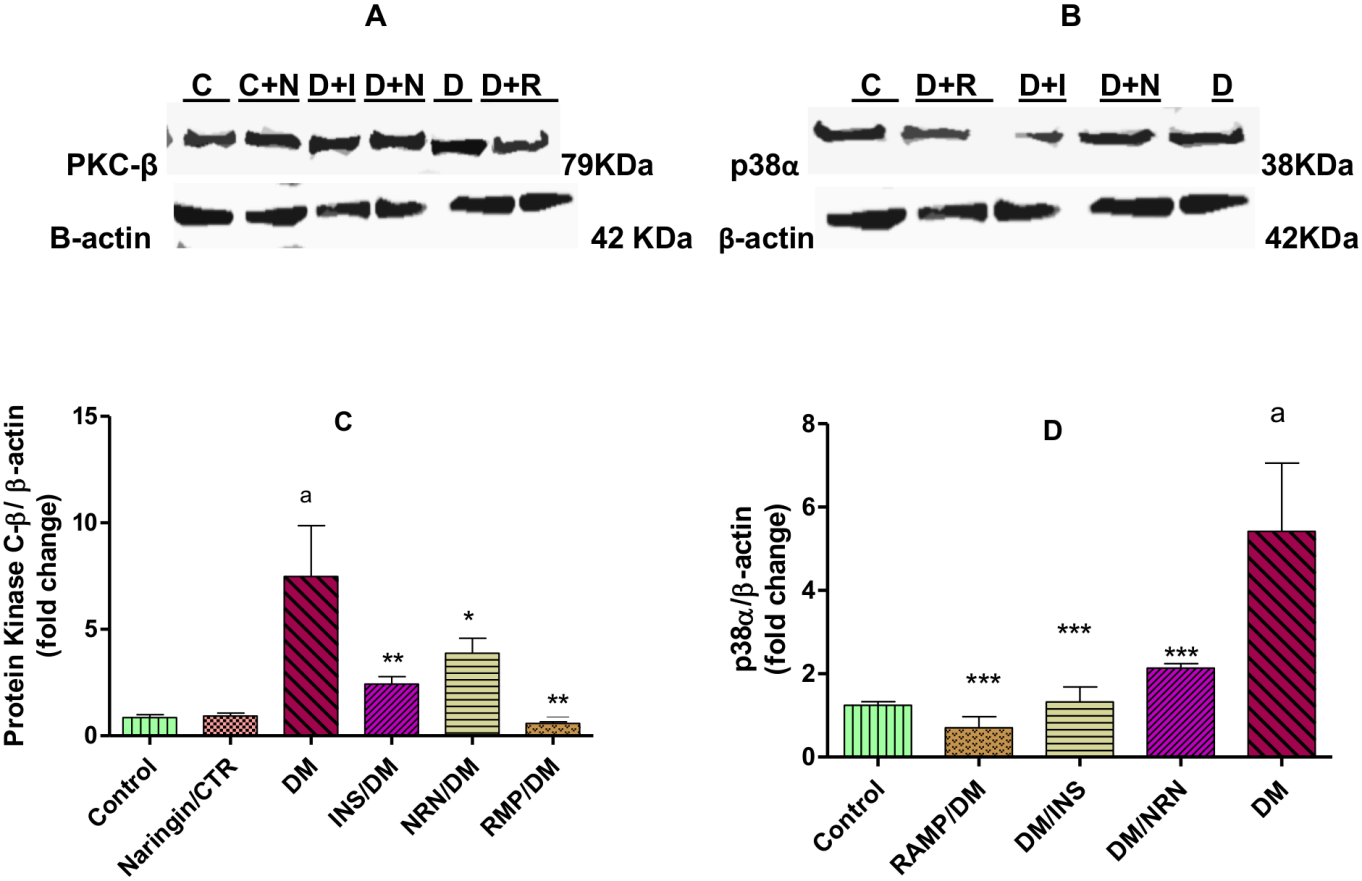
Immunoblot assays of **A**: Protein kinase C-β and **B**: p38α protein expressions in the myocardium. Densitometry scans of immunoblots of **C**: Protein Kinase C-β and **D**: p38α protein expression in the myocardium. [*p<0.05, **p<0.01 (compared to DM) and ^a^p<0.001 (compared to CTR)]. Control (CTR or C), naringin (NRN or C+N), insulin (INS or D+I) diabetes mellitus (DM or D), Diabetes/naringin (D+N) and ramipril (RMP or D+R).

There were markedly significant (p<0.001) 7-fold increases in the expression of PKC protein in the myocardium of untreated diabetic rats compared to the vehicle-treated non-diabetic controls (Fig 7A and 7C). However, naringin, insulin and ramipril treatment of diabetic rats significantly (p<0.05) reduced protein kinase C expression by 30%, 67% and 80% relative to untreated diabetic controls, respectively. The untreated diabetic rats showed a significant (p<0.001) 5-fold increase in the myocardial expression of p38 MAPK in the myocardium compared to the vehicle-treated non-diabetic controls (Fig 7B and 7D). However, treatment of the diabetic rats with naringin, insulin and ramipril resulted in significant (p<0.01) reduction (64%, 68% and 73%, respectively) in protein kinase C expression relative to the untreated diabetic controls.

## Discussion

Myocardial fibrosis constitutes one of the principal structural changes in a diabetic heart which frequently occurs in diabetic cardiomyopathy and is an important predisposing factor to the development of heart failure in diabetic patients [1]. Hyperglycemia is a principal promoter of myocardial fibrosis in diabetic patients and experimental animals [2, 3, 7, 8]. In this study, we successfully created a type 1 diabetes model as evidenced by observed significant hyperglycemia, glucose intolerance, polydipsia, polyuria weight loss and hypoinsulinemia (unpublished data) coupled with significantly increased fibrotic areas in the myocardium of untreated diabetic rats. Our findings of increased fibrotic tissues in the hearts of diabetic rats coupled with marked hyperglycemia suggest that the profibrotic events in the diabetic hearts were hyperglycemia driven. This is consistent with previous findings by other investigators that have reported increased fibrotic scars in the hearts of diabetic patients and experimental animals [2, 3]. Furthermore, naringin treatment significantly reduced percentage fibrotic area by 67% compared to untreated diabetic rats suggesting that it has significant anti-fibrotic properties (Figs 5 and 6). Naringin supplementation has previously been shown to improve diet-induced cardiovascular dysfunction in experimental animals [25]. However, FBG and oral GTTs suggested that naringin did not exert its anti-fibrotic effects by lowering blood glucose concentrations which is consistent with previous reports that naringin does not exert significant hypoglycemic effects in type 1 diabetes although its anti-hyperglycemic activity are pronounced in type 2 diabetes mellitus [22, 23]. It is therefore likely that anti-fibrotic effects of naringin reported in this study occurred independently of blood glucose concentrations.

In order to understand the mechanism(s) by which naringin exerted its anti-fibrotic effects, we investigated the effects of naringin on NADPH oxidase activity in the myocardium. NADPH oxidase over-activity in diabetes has been identified as one the principal mechanisms by which hyperglycemia promotes its pro-fibrotic effects [9–11]. There were significantly increased NADPH oxidase activities in the diabetic rats (Fig 2) in concurrence with previously reported cases [3, 9–11]. In our study, naringin significantly and modestly reduced cardiac tissue NADPH oxidase activity suggesting a reduction of ROS in the myocardium of diabetic rats. Previously, ROS enhancement of NADPH oxidase activity has been reported, therefore it is likely that naringin through its antioxidant actions reduced NADPH oxidase activity by reducing myocardial ROS concentrations. To confirm this we indirectly determined ROS production in the myocardium by measuring protein products of oxidative damage which were significantly increased in diabetic rats suggesting increased oxidative stress arising from increased oxidant generation coupled with reduced CuZnSOD activity. Increased oxidant species generation and antioxidant depletion in myocardial cells under conditions of hyperglycemia have previously been reported [2, 31, 32]. Naringin, significantly reduced oxidized protein products both in the plasma and the cardiac tissues confirming that it reduced myocardial oxidant stress possibly through it antioxidant properties as previously reported [16, 17, 24]. Naringin also significantly increased CuZnSOD activity in diabetic rats suggesting that apart from its radical scavenging activities, it also exerts antioxidant effects by increasing the CuZnSOD activity in the cardiac tissue. Naringin is a known hydroxyl and superoxide radicals scavenger [33] and has previously been demonstrated to boost antioxidant enzymes such as SOD, catalase and GPx content in diabetic animals [34, 35]. Furthermore, naringin was shown to prevent formation of lipid peroxidation products and hydroperoxides in plasma and hearts of isoproterenol-induced cardiotoxicity in Wistar rats [36].

This narrative is supported by the fact that ROS is known to activate cardiac fibroblasts which significantly contribute to pro-fibrotic events through increased extracellular matrix remodeling [9–11]. Furthermore, extracellular matrix remodeling is associated with increased cardiac fibroblast proliferation and activation, events that have been shown to be influenced by redox state of the cells [9–11]. It is therefore possible that naringin by mopping up ROS limits profibrotic events through inactivation and reduction of cardiac fibroblast proliferation.

Hyperglycemia-associated pro-fibrotic effects are associated with the up-regulation and activation of signaling molecules such as PKC and mitogen activated kinases such as c-Jun Nuclear Kinase (JNK), p38 MAPK and extracellular signal related kinase 1 (ERK1/2) which contribute to adverse remodeling in the diabetic hearts [3]. In this study, we observed significant up-regulation of PKC-β expression in the untreated diabetic rats which have previously been shown and suggested to be due to hyperglycemia-induced increases in diacylglycerol (DAG), a known activator of PKC [3]. PKC-β up-regulation has been previously shown to be associated with pro-fibrotic gene expression (TGF-β1, CTGF and PAI-1) leading to increased myocardial fibrosis [3]. PKC is also known to activate NADPH oxidase activity leading to increases in ROS production which triggers PKC activation leading to further pro-fibrotic events [3]. Naringin significantly down-regulated PKC-β1 activity suggesting that some of its anti-fibrotic effects are due to down-regulation of PKC expression. It is likely that naringin’s probable mechanism of down-regulating PKC-β might be associated with the positive feedback actions of increased myocardial tissue ROS on PKC. By reducing myocardial ROS, the activation of PKC-β was reduced hence the decrease in the expression of PKC-β in the cardiac tissue.

Furthermore there was up-regulation of p38α in the untreated diabetic rats compared to the nondiabetic controls. However, treatment of diabetic rats with naringin down-regulated the expression of p38α in diabetic rats suggesting that some of naringin’s anti-fibrotic activity might be mediated by down-regulation of p38α. NADPH oxidase, ROS and PKC are important factors in the up-regulation and activation of p38α [3]. Considering that increased oxidative stress has been described as potent pro-fibrotic factor and the established antioxidant actions of naringin, it is likely that naringin through its antioxidant effects limits ROS burden within the myocardium, and possibly leads to down-regulation of p38α. These effects of naringin may further be potentiated by its demonstrated anti-inflammatory effects. Naringin supplementation has been associated with reduced inflammation and fibrosis in isoproterenol-treated [36] and also in high fat diet-fed rats [25]. Although we did not measure cardiac expression of pro-inflammatory cytokines, our results presented here appear to resonate with previous studies that suggested a downregulation of the expression of TNF-α, TGF-β1, matrix metalloproteinase 1 and tissue inhibitor metalloproteinase 1 which are known regulators of fibrosis in tissues [37]. These anti-inflammatory effects could have been mediated in part by down-regulation of p38α and PKC-β1 that we have shown in our results, respectively. Cardiomyocyte protection against hyperglycemia-induced phosphorylation and activation of p38 and p53 proteins and ROS-activated MAPK signaling pathways by nairingin have previously been demonstrated [37, 38].

## Conclusion

Naringin exerts anti-fibrotic effects by inhibition of oxidative stress, NADPH oxidase activity, and the down-regulation of PKC-β and p38α. Therefore naringin may be useful in mitigating the effects of cardiac fibrosis in type 1 diabetes through its antioxidant effects and therefore limit cardiac remodeling.

## Supporting Information

S1 Appendix Fasting blood glucose concentrations±SD (mM) at baseline (Day 0) and at the end of the study (Day 56). (PDF)

S2 Appendix Average water consumption (ml/day) during treatment period. (PDF)

S3 Appendix Calulates Areas Under The Curce (AUC) from Glucose Tolerance Tests (GTT) and expressed as AUC units. (PDF)

S4 Appendix Calculated differences in live body weights (g) at baseline and at the end of the experiments. (PDF)

S5 Appendix NADPH activity expressed as nmol/mg protein. (PDF)

S6 Appendix CUZnSOD activity expressed as (U/ml). (PDF)

S7 Appendix Plasma and cardiac Advanced Oxodation Protein Products (AOPP) concentrations expressed as chloramine-T equivalents. (PDF)

S8 Appendix Calculated percentage fibrosis of the cardiac tissue. (PDF)

S9 Appendix Absolute values measured and used as confirmation of diabetes (Table A) and as markers of oxdative stress and cardiac fibrosis (Table B), respectively. (DOCX)
