# Supplementary material for: Naringin Reduces Hyperglycemia-Induced Cardiac Fibrosis by Relieving Oxidative Stress
Source: PLoS One. 2016 Mar 11;11(3):e0149890. doi: 10.1371/journal.pone.0149890 (PMC4788433; doi:10.1371/journal.pone.0149890)
Supplement: S1 Appendix — (PDF) [file pone.0149890.s001.pdf]

S1

	Day 0		no		Day 56	no
Control	4.09	0.18	7	5.34	0.25	7
Control + Naringin	4.13	0.19	7	4.97	0.41	7
DM +INS	4.14	0.19	7	16.91	0.97	7
DM + NRN	3.83	0.26	7	33.30	0.45	7
DM	3.93	0.17	7	34.61	0.76	7
DM+RAMP	4.21	0.26	7	30.77	1.78	7
