# Supplementary material for: Naringin Reduces Hyperglycemia-Induced Cardiac Fibrosis by Relieving Oxidative Stress
Source: PLoS One. 2016 Mar 11;11(3):e0149890. doi: 10.1371/journal.pone.0149890 (PMC4788433; doi:10.1371/journal.pone.0149890)
Supplement: S2 Appendix — (PDF) [file pone.0149890.s002.pdf]

S2

Water consumption-

CTR	NRN/Norm.	INS/DM	NRN/DM	DM	DM/RAMP
275.	260.	1050.	1060.	1180.0	1140.
225.	250.	1072.	820.	1070.0	850.
300.	225.	1050.	1225.	1250.0	765.
314.	240.	1250.	1095.	1170.0	920.
370.	250.	1000.	1110.	1120.0	1090.
260.	225.	1140.	1375.	1225.0	1075.
350.	200.	1150.	1175.	1125.0	1000.
210.	200.	1200.	1225.	1265.0	1075.
212.	225.	1275.	1175.	1100.0	1000.
250.	175.	1225.	1125.	1075.0	1120.
230.	250.	1320.	1135.	1035.0	880.
225.	200.	980.	1065.	1025.0	850.
235.	185.	795.	1040.	1050.0	800.
150.	125.	1345.	850.	650.0	750.
250.	195.	1090.	1000.	945.0	800.
175.	250.	1400.	900.	950.0	800.
350.	275.	1365.	900.	860.0	865.
260.	200.	1225.	775.	840.0	715.
205.	250.	1135.	900.	715.0	565.
200.	325.	1100.	875.	740.0	710.
175.	215.	960.	900.	1050.0	700.
275.	200.	860.	900.	1250.0	750.
175.	190.	790.	975.	1025.0	585.
230.	200.	840.	1010.	1330.0	545.
180.	205.	735.	870.	1425.0	515.
210.	175.	710.	915.	1450.0	425.
200.	175.	625.	800.	1462.5	750.
305.	315.	825.	800.	1350.0	520.
225.	175.	700.	670.	1510.0	410.
225.	200.	575.	700.	1500.0	575.
175.	175.	775.	785.	1520.0	380.
205.	160.	655.	730.	1430.0	480.
175.	200.	725.	775.	1475.0	530.
200.	225.	775.	585.	1415.0	450.
225.	185.	790.	700.	1415.0	570.
135.	220.	350.	475.	1590.0	400.
