# Supplementary material for: Naringin Reduces Hyperglycemia-Induced Cardiac Fibrosis by Relieving Oxidative Stress
Source: PLoS One. 2016 Mar 11;11(3):e0149890. doi: 10.1371/journal.pone.0149890 (PMC4788433; doi:10.1371/journal.pone.0149890)
Supplement: S3 Appendix — (PDF) [file pone.0149890.s003.pdf]

S3

Glucose Tolerance Test

Control	Control+NRN	INS/DM	DM+NRN	DM	DM+RAMP
791.3	984.8	3939.	3740.	3897.	4034.
900.8	804.8	3788.	3962.	3952.	4032.
861.0	972.8	3876.	3941.	3505.	4043.
902.3	849.0	3917.	3877.	3459.	4038.
859.5	1007.0	3870.	3414.	3717.	4036.
1176.0	895.5	3835.	3401.	3789.	3039.
	951.0	3219.	3983.		
