# Supplementary material for: Naringin Reduces Hyperglycemia-Induced Cardiac Fibrosis by Relieving Oxidative Stress
Source: PLoS One. 2016 Mar 11;11(3):e0149890. doi: 10.1371/journal.pone.0149890 (PMC4788433; doi:10.1371/journal.pone.0149890)
Supplement: S4 Appendix — (PDF) [file pone.0149890.s004.pdf]

S4

Weight Change

CTR	NRN/Norm.	INS/DM	NRN/DM	DM	DM/RAMP
88.	86.	71.	-31.	-57.	-65.
71.	119.	44.	5.	14.	
141.	112.	14.	3.		-43.
100.	88.	54.	-21.	-68.	-14.
95.	113.	53.	-5.	-73.	-27.
110.	107.	55.	-59.	-33.	-55.
138.	87.	91.	-46.	-52.	-44.
