# Supplementary material for: Naringin Reduces Hyperglycemia-Induced Cardiac Fibrosis by Relieving Oxidative Stress
Source: PLoS One. 2016 Mar 11;11(3):e0149890. doi: 10.1371/journal.pone.0149890 (PMC4788433; doi:10.1371/journal.pone.0149890)
Supplement: S5 Appendix — (PDF) [file pone.0149890.s005.pdf]

S5

NADPH Oxidase activity

CTR	NRN+CTR	INS+DM	NRN+DM	DM	RMP+DM
0.314	0.4760	0.286	0.91620	1.330	0.143
0.190	0.2100	0.048	0.83810	1.240	0.095
0.224	0.4500	0.143	1.31900	0.910	0.524
0.333	0.3620	0.076	0.73300	1.290	0.429
0.476	0.3500	0.238	0.84800	1.000	0.429
