# Supplementary material for: Naringin Reduces Hyperglycemia-Induced Cardiac Fibrosis by Relieving Oxidative Stress
Source: PLoS One. 2016 Mar 11;11(3):e0149890. doi: 10.1371/journal.pone.0149890 (PMC4788433; doi:10.1371/journal.pone.0149890)
Supplement: S6 Appendix — (PDF) [file pone.0149890.s006.pdf]

S6

CuZnSOD activity

CTR	NRN	INS	NRN/DM	DM	RMP/DM
1.125929	1.736223	1.367032	0.7002298	0.2858327	0.3159707
1.864309	1.318058	1.272851	0.5457727	0.2029533	0.229324
1.807800	1.355731	1.174903	0.5909797	0.3385742	0.5231692
1.099558	1.706084	1.687248	0.7039971	0.1577464	0.1652808
1.054351	1.416007	1.092023	1.084489	0.2933672	0.4666606
		1.717386		0.5269365	0.2519275
				0.6550228	
