# Supplementary material for: Naringin Reduces Hyperglycemia-Induced Cardiac Fibrosis by Relieving Oxidative Stress
Source: PLoS One. 2016 Mar 11;11(3):e0149890. doi: 10.1371/journal.pone.0149890 (PMC4788433; doi:10.1371/journal.pone.0149890)
Supplement: S7 Appendix — (PDF) [file pone.0149890.s007.pdf]

S7

1. Plasma AOPP-

CTR	CTR+NRN	DM+INS	DM+NRN	DM	DM+RAMP
5995.508	6740.368	7711.001	8071.853	8801.275	6647.742
7095.852	5982.000	6690.196	5518.875	9785.704	7153.321
6739.946	7108.938	7180.336	8475.157	8602.384	6584.063
6063.047	6609.148	8002.384	8087.290	8361.306	7095.430
5779.382	6535.820	8245.524	7846.079	8210.790	7859.586

2. Cardiac AOPP-

CTR	NRN	INS/DM	NRN/DM	DM	DM/RMP
156.7445	143.4215	150.9753	135.7252	182.9474	159.6693
135.1551	146.2720	156.9613	135.2976	171.8305	144.2767
155.8212	153.2557	163.0434	142.4239	186.7490	143.5641
154.1109	164.0876	161.2371	117.3395	162.8049	140.4285
154.9660	160.3819	160.6670	107.2203	158.6717	161.6647
