# Supplementary material for: Naringin Reduces Hyperglycemia-Induced Cardiac Fibrosis by Relieving Oxidative Stress
Source: PLoS One. 2016 Mar 11;11(3):e0149890. doi: 10.1371/journal.pone.0149890 (PMC4788433; doi:10.1371/journal.pone.0149890)
Supplement: S8 Appendix — (PDF) [file pone.0149890.s008.pdf]

S8

Percentage Fibrosis

CTR	NRN	DM/INS	DM/NRN	DM	DM/RMP
1.975190	3.698875	6.352830	8.121321	42.861210	8.761732
2.623611	3.748647	13.591230	5.609208	45.512740	24.924660
2.952600	2.024492	7.427301	8.996595	29.196830	12.195290
3.535379	2.488781	25.299670	5.429354	51.068450	4.642890
2.789490	3.207859	24.419670	7.783926	52.545640	11.380050
