# Supplementary material for: Naringin Reduces Hyperglycemia-Induced Cardiac Fibrosis by Relieving Oxidative Stress
Source: PLoS One. 2016 Mar 11;11(3):e0149890. doi: 10.1371/journal.pone.0149890 (PMC4788433; doi:10.1371/journal.pone.0149890)
Supplement: S9 Appendix — (DOCX) [file pone.0149890.s009.docx]

**Table A:** Confirmation of diabetes mellitus

|  | **Control** | **Control + Naringin** | **Diabetes + Insulin** | **Diabetes + Naringin** | **Diabetes** | **Diabetes +Ramipril** |
| --- | --- | --- | --- | --- | --- | --- |
| Final FBG (mmol/L) | 5.70±0.25 | 4.97±0.41 | 16.91±0.97^b^ | 33.30±0.45 | 34.61±0.76^a^ | 30.71±1.78 |
| Initial FBG (mmol/L) | 4.09±0.18 | 4.13±0.14 | 4.14±0.20 | 3.83±0.26 | 3.93±0.17 | 4.21±0.26 |
| AUC (x10^3^) | 0.92±0.55 | 0.92± 0.29 | 3.78±0.95^b^ | 3.76±0.96^b^ | 3.72±0.83^a^ | 3.87±0.17^b^ |
| Water Consumption (L)/day | 0.23±0.01 | 0.21±0.01 | 0.97±0.04^b^ | 0.92±0.03^b^ | 1.18±0.04^a^ | 0.73±0.04^b^ |
| Weight Change (g) | 106.1±9.72 | 102±5.37 | 54.6±8.94^b^ | -22±9.35^b^ | -44.8±13.1^a^ | -41.3±7.55^b^ |

^a^p<0.05 (compared to non-diabetic controls) and ^b^p<0.05 (compared to untreated diabetic rats)

**Table B**: Oxidative parameters and percentage area of fibrosis in myocardium

|  | **Control** | **Control + Naringin** | **Diabetes + Insulin** | **Diabetes + Naringin** | **Diabetes** | **Diabetes +Ramipril** |
| --- | --- | --- | --- | --- | --- | --- |
| Plasma AOPP (x10^3^) | 6.34±0.25 | 6.60±0.18 | 7.57±0.28^b^ | 7.60±0.53^b^ | 8.75±0.28^a^ | 7.07±0.23^b^ |
| Cardiac AOPP (x10^3^) | 0.15±0.004 | 0.15±0.004 | 0.16±0.002^b^ | 0.13±0.007^b^ | 0.17±0.005^a^ | 0.15±0.004^b^ |
| NADPH Oxidase activity (mmol/mg protein) | 0.31±0.05 | 0.37±0.05 | 0.16±0.05^b^ | 0.93±0.10^b^ | 1.15±0.08^a^ | 0.32±0.09^b^ |
| CuZnSOD (U/ml) | 1.39±0.18 | 1.51±0.09 | 1.39±0.11^b^ | 0.73±0.09^b^ | 0.35±0.07^a^ | 0.33±0.06 |
| Fibrotic Area (%) | 2.78±0.25 | 3.03±0.34 | 15.42±4.05 | 7.19±0.71 | 49.24±4.16 | 12.38±3.40 |

^a^p<0.05 (compared to non-diabetic controls) and ^b^p<0.05 (compared to untreated diabetic rats)
